# The first record of *Calamaria nebulosa* Lee, 2021 (Squamata, Calamariidae) from Yunnan Province, China and an expanded morphological description based on newly-collected specimens

**DOI:** 10.3897/BDJ.14.e217560

**Published:** 2026-11-05

**Authors:** Guocheng Shu, Chunmei Fu, Jun Huang, Cheng Li, Zhongping He, Peng Guo, Bingjun Dong, Shun Ma

**Affiliations:** 1 Faculty of Agriculture, Forestry and Food Engineering, Yibin University, Yibin, China Faculty of Agriculture, Forestry and Food Engineering, Yibin University Yibin China; 2 Yibin Key Laboratory of Zoological Diversity and Ecological Conservation & Yibin Inspection and Quarantine Engineering Technology Research Center of Animal and Plant, Yibin University, Yibin, China Yibin Key Laboratory of Zoological Diversity and Ecological Conservation & Yibin Inspection and Quarantine Engineering Technology Research Center of Animal and Plant, Yibin University Yibin China; 3 College of Life Sciences, Shenyang Normal University, Shenyang, China College of Life Sciences, Shenyang Normal University Shenyang China; 4 Chengdu Institute of Biology, Chinese Academy of Sciences, Chengdu, China Chengdu Institute of Biology, Chinese Academy of Sciences Chengdu China; 5 University of Chinese Academy of Sciences, Beijing, China University of Chinese Academy of Sciences Beijing China; 6 Hunan Normal University, Changsha, China Hunan Normal University Changsha China

**Keywords:** morphology, mtDNA, new national record, phylogeny, reed snakes

## Abstract

**Background:**

*Calamaria nebulosa* Lee, 2021 was originally described, based on a single female holotype collected in Phongsaly Province, Laos. Until now, this species has been known only from a single specimen and exhibits an extremely restricted geographic distribution. Consequently, its intraspecific morphological variation and biogeography remain poorly understood. Several *Calamaria* specimens collected during recent field surveys were identified as *C. nebulosa* using morphological and molecular data, representing a new national record for China.

**New information:**

Genetic analyses, based on the mitochondrial cytb gene, confirmed that these specimens clustered within a well-supported *C. nebulosa* clade, with uncorrected *p*-distances of 1.2%–1.3% relative to the holotype. Herein, we provide a detailed morphological description of Chinese *C. nebulosa* specimens and revise the species diagnosis: rostral scale wider than high; the portion of rostral scale visible in dorsal view is approximately half the length of the prefrontal suture; six scales and shields surround the paraparietal scale; preocular scale absent; four supralabials, with the second and third touching the orbit; five infralabials, the first pair separating the mental from the anterior chin-shields; posterior chin-shields meeting only anteriorly; five indistinct dark-brown longitudinal dorsal stripes; ventral surface of tail uniformly yellow and immaculate in preservative; distinct white nuchal collar absent; dorsal scales reduce from six to four rows between the last 1^st^ and 4^th^ subcaudal scales; ventrals scales 179-192 in males and 198-206 in females. To date, a total of twelve *Calamaria* species have been recorded from China.

## Introduction

*Calamaria* Boie, 1827 (Calamariidae) is a genus of small-sized and semi-fossorial snakes widely distributed across the Oriental Realm (Weinell et al. 2020, Korolev et al. 2026, Lalremsanga et al. 2026). Morphologically, species of this genus are characterised by a cylindrical body with an indistinct head-neck boundary; the head lacks internasal, loreal and temporal scales; the dorsal scales are smooth, with 13 rows anteriorly and at mid-body; and they possess undivided anal scales and divided subcaudal scales (Zhao 2006, Zhang et al. 2026). Due to highly conserved external morphology and cryptic microhabitat preferences, interspecific delineation within *Calamaria* has long been taxonomically challenging (Qi et al. 2026a). India, China and mainland Southeast Asia represent a key diversification centre for *Calamaria*. In recent years, integrative taxonomic approaches, combining rigorous scalation comparisons, morphometric assessments and molecular data, have profoundly advanced the systematics of *Calamaria* in these areas (Aksornneam et al. 2026, Qi et al. 2026b). For example, several populations formerly assigned to *C. pavimentata* Duméril, Bibron & Duméril have been described as new species, including *C. arcana* Yeung, Lau & Yang, 2022, *C. flavicollaris* Aksornneam, Rujirawan, Yodthong, Pawangkhanant, Sung & Aowphol, 2026, *C. garoensis* Bharali, Sangma, Amarasinghe, Bohra, Swargiary, Marak, Hazarika, Das, Asem, Lyngdoh, Lalremsanga & Purkayastha, 2026, *C. mizoramensis* Lalremsanga, Malsawmdawngliana, Bal, Abeiryureilai, Hruaia, Korolev, Vogel, Poyarkov & Nguyen, 2026, *C. synergis* Zhang, Xu, Nguyen, Poyarkov, Vogel, Wang & Huang, 2025, *C. similis* Qi, Nguyen, Yang, Xu, David, Shi, Liu, Rong, Korolev & Wang, 2026, *C. hunanensis* Zhang, Zhu, Wei, Shu, Li, Gernot, Niu, Ding, Chen, Guo & Shi and *C. incredibilis* Qi, Nguyen, Wang, Zeng, Shi, Xu, Song, Huang, Mo, Poyarkov & Wang, 2026 (Yeung et al. 2022, Zhang et al. 2025, Aksornneam et al. 2026, Bharali et al. 2026, Lalremsanga et al. 2026, Qi et al. 2026a, Qi et al. 2026b, Zhang et al. 2026). *C. nebulosa* Lee, 2021 was formerly treated as *C. yunnanensis* Chernov, 1962, before being formally described as a valid species (Lee 2021).

*C. nebulosa* is a poorly-documented species with a narrow distribution and scarce voucher specimens (Lee 2021). Its intraspecific morphological variation, geographic range boundaries and regional faunal affinities remain insufficiently clarified. During recent field surveys, several newly-collected *Calamaria* specimens from China were identified as *C. nebulosa*, based on morphological and molecular evidence, representing a new national record for China.

This study reports new distribution records of *C. nebulosa* from Yunnan Province, China and updates diagnostic traits of adult males and females. Our findings extend the known distribution range of *C. nebulosa*, augment documented reptile diversity of Yunnan and provide baseline data for future biogeographic studies and conservation evaluation of semi-fossorial snakes from subtropical south-western China.

## Materials and methods

### Sampling

We collected three road-killed specimens of *C. nebulosa* from Simao District, Pu'er City, Yunnan Province, China (Fig. 1). Liver or muscle tissue samples were preserved in 95% ethanol and stored at -80℃ for molecular analyses. All specimens were deposited in the Yibin Key Laboratory of Zoological Diversity and Ecological Conservation, Yibin University, Sichuan Province, China.

### Molecular analyses

Total genomic DNA was extracted from muscle or liver tissue using the MolPure Cell/Tissue DNA Kit (YEASEN). A fragment of the mitochondrial cytochrome b (cyt *b*) gene was amplified using the primer pair L14910/H16064 (Burbrink et al. 2000). The total volume of the PCR reaction mixture was 25 μl, consisting of 9.5 μl ddH_2_O, 1 μl of forward primer, 1 μl of reverse primer, 12.5 μl 2× Hieff PCR Master Mix (with dye, YEASEN) and 1 μl template DNA. PCR amplification was performed under the following thermal cycling conditions: an initial denaturation step at 94℃ for 5 min; followed by 35 cycles of 94℃ for 30 s, 48℃ for 45 s and 72℃ for 1 min; and a final extension step at 72℃ for 5 min. PCR product purification and bidirectional Sanger sequencing were completed by Sangon Biotech.

All sequences were assembled and manually edited using SeqMan (Lasergene v.15.1, DNASTAR, USA). The molecular dataset comprised three new cyt *b* sequences of *C. nebulosa*, 53 congeneric sequences representing 21 nominal *Calamaria* species and three outgroup taxa, including *Orientocoluber spinalis*, *Elaphe quatuorlineata* and *Lycodon rufozonatus* (Suppl. material 1). Outgroups were selected, based on previous phylogenetic studies to maintain taxonomic consistency. Sequence alignment was performed using the MUSCLE in MEGA 7.0.26 (Kumar et al. 2016).

We used PartitionFinder v.2.1.1 (Lanfear et al. 2016) to select the partitioning schemes and best-fit substitution models for Bayesian Inference (BI). BI analyses were run in MrBayes v.3.2 (Ronquist et al. 2012). Three independent searches were conducted, each initiated with a random tree and four Markov chains (three heated and one cold) for 10×10^6^ generations, with the first 25% of samples discarded as burn-in. Effective sample sizes (ESS) were inspected in Tracer v.1.7.2 (Rambaut et al. 2018). The nodal support of the BI tree was expressed as Bayesian posterior probabilities (PP), with PP ≥ 0.95 regarded as good support (Larget and Simon 1999). The Maximum Likelihood (ML) and best nucleotide substitution model analyses was calculated by IQ-TREE v.2.1.3 (Chernomor et al. 2016) and 1000 ultrafast bootstrap (UFB) were generated to assess node support (Hoang et al. 2017). Branches with bootstrap support (BS) ≥ 75 were considered well supported (Hillis and Bull 1993). Trees were visualised in FigTree v.1.4 (Rambaut 2012). Pairwise uncorrected p-distances between lineages were calculated in MEGA v.7.0.26 (Kumar et al. 2016).

### Morphological analysis

Terminology and measurement methods referred to the relevant contents of Zhao (2006), Poyarkov et al. (2019) and Lee (2021). Sex was determined by dissecting for hemipenes and inferred from relative tail length. Ventral scales counts followed Dowling (1951). Morphological characters of the examined specimens were compared in detail with holotype of FMNH 258666 (Lee 2021). Abbreviations for the measured morphological characters are provided in Suppl. material 2.

## Taxon treatments

### Calamaria nebulosa

Lee, 2021

008E1716-D82E-5E2F-9B3D-D5DA1C1169F4

13AC3177-FFBD-4C12-9132-A9BA5ED7BB48

#### Materials

- **Type status:** Other material. **Occurrence:** catalogNumber: MS856; recordedBy: Cheng Li and Tao Yang; individualCount: 1; sex: male; lifeStage: adult; **Taxon:** scientificName: Calamaria nebulosa; kingdom: Animalia; phylum: Chordata; class: Reptilia; order: Squamata; family: Calamariidae; scientificNameAuthorship: Lee, 2021; **Location:** country: China; countryCode: CN; stateProvince: Yunnan; county: Pu'er City; locality: Simao District; verbatimElevation: 1452 m; verbatimCoordinateSystem: 22.774026 °N 101.18377 °E; **Event:** eventDate: 24-06-2020; **Record Level:** basisOfRecord: Preserved Specimen
- **Type status:** Other material. **Occurrence:** catalogNumber: MS857; recordedBy: Cheng Li and Tao Yang; individualCount: 1; sex: female; lifeStage: adult; **Taxon:** scientificName: Calamaria nebulosa; kingdom: Animalia; phylum: Chordata; class: Reptilia; order: Squamata; family: Calamariidae; scientificNameAuthorship: Lee, 2021; **Location:** country: China; countryCode: CN; stateProvince: Yunnan; county: Pu'er City; locality: Simao District; verbatimElevation: 1447 m; verbatimCoordinateSystem: 22.774629 °N 101.101378 °E; **Event:** eventDate: 24-06-2020; **Record Level:** basisOfRecord: Preserved Specimen
- **Type status:** Other material. **Occurrence:** catalogNumber: MS858; recordedBy: Cheng Li and Tao Yang; individualCount: 1; sex: female; lifeStage: adult; **Taxon:** scientificName: Calamaria nebulosa; kingdom: Animalia; phylum: Chordata; class: Reptilia; order: Squamata; family: Calamariidae; scientificNameAuthorship: Lee, 2021; **Location:** country: China; countryCode: CN; stateProvince: Yunnan; county: Pu'er City; locality: Simao District; verbatimElevation: 1452 m; verbatimCoordinateSystem: 22.773236 °N 101.100528 °E; **Event:** eventDate: 24-06-2020; **Record Level:** basisOfRecord: Preserved Specimen

#### Description

##### Measurements

All specimens are small-bodied snakes with an elongate body form. Male specimen (YBU-MS856, Fig. 2): SVL 275.5 mm; TailL/TotalL ratio 0.089; Mid-body diameter 3.37 mm; Tail base diameter 3.59 mm. Female specimen (YBU-MS857, Fig. 3; YBU-MS858, Fig. 4): SVL 256.0 mm and 359.7 mm; TailL/TotalL ratio 0.056 and 0.079; Mid-body diameter 3.04 mm and 4.71 mm; Tail base diameter 2.32 mm and 3.65 mm.

##### Head morphology and scalation

Head is small and ovate in dorsal view, without clear demarcation from the neck. All newly-collected specimens lack internasal, loreal, temporal, preocular, interparietal and occipital scales. Snout is blunt. Nostrils are small and elliptical. Eyes small and elliptical; eye diameter is shorter than naris-eye distance. Rostral scale is triangular in dorsal view and wider than tall; the suture between prefrontal and rostral scale forms a shallow V shape. Nasal scales are small. Prefrontal are longer than wide and slightly shorter than the frontal scale. Frontal scale is subhexagonal and length-to-width ratio ranges from 1.06 to 1.42. The anterior angle formed by the suture is greater than 90°, whereas the posterior angle formed by the parietal suture is less than 90°. Parietal scales are longer than wide, broader anteriorly narrower posteriorly and longer than the frontal scale. Paraparietal scales contact the parietal scale and are surrounded by six scales. Supraocular scales are longer than wide. Postocular scales are broader than long. Supralabial scale four; the 1^st^ and 3^rd^ are the smallest and 4^th^ is largest and contacts postocular and parietal scales; the 1^st^ in contacts the nasal scale, the 2^nd^ and 3^rd^ in contact the eye. Infralabial scales five; the 1^st^ is smallest and prevents the mental scale from contacting anterior chin-shield scales; the 1^st^ to 3^rd^ contact anterior chin-shield scales; 3^rd^ and 4^th^ contact posterior chin-shield scales; 5^th^ is largest. Mental scale is triangular and wider than long. Anterior chin-shields are paired, pentagonal or subrectangular and in full medial contact with each other. Posterior chin-shields are paired and subtriangular and are separated medially by the first preventral gular scale.

##### Body scalation

Dorsal scale rows are 13–13–13 throughout body and all dorsal scales are smooth. Ventral scales counts 179–206. The anal scale is undivided and slightly larger than a ventral scale.

##### Tail scalation

All subcaudal scales are paired. End of tail obtuse point. Dorsal scale rows reduce from 13 to 8 at the 1^st^ subcaudal scale in both sexes; from 8 to 6 rows at the 4^th^ subcaudal scale in both sexes; from six to four rows at last 4^th^ subcaudal scale in the male and at last 1^st^, 2^nd^ and 4^th^ subcaudal scale in females.

##### Colouration in preservative

After formalin fixation and storage for 5 years in 75% ethanol, the three newly-collected specimens were dark brown dorsally. Dorsal scale margins are dark-brown; five longitudinal stripes originate at the nape and fade posteriorly towards the tail; top of head same colour as dorsum, faint yellow patterns on the occipital part of the head; bottom half of supralabials and rostral pale yellow; underside of head and rest of venter also pale yellow, margins of ventral scales immaculate; underside of tail the same as rest of venter. The scales of posterior body and tail exhibit structural colouration with blue under intense light. Colour in life not recorded.

#### Diagnosis

##### Revised diagnosis

Morphological features of the three examined specimens are consistent with the original species description of *C. nebulosa* (Table 1), as follows: (1) Rostral scale wider than high; (2) The dorsally visible portion of the rostral scale is roughly half the length of the prefrontal suture; (3) Six scales and shields surrounding the paraparietal scale; (4) Preocular scale absent; (5) Four supralabials, with the 2^nd^ and 3^rd^ touching the orbit; (6) Five infralabials, the first pair separating the mental scale from the anterior chin shields; (7) Distinct white nuchal collar absent; (8) Posterior chin-shields meeting only anteriorly; (9) ventrals scales 179-192 in males and 198-206 in females.(10) Five faint dark brown longitudinal stripes on dorsum; (11) Ventral side of tail uniformly yellow in preservative.

##### Morphological variation

Compared with the holotype, major variation was observed in ventrals scale counts 192-206 (vs. 179), subcaudals scale counts 17-23 (vs. 22), DSR6R at subcaudals 4^th^ (vs. 12^th^), DSR4R at subcaudals 14^th^-20^th^ (vs. 22^th^) and dorsal coloration in preservative dark-brown (vs. bluish-grey).

##### Comparisons

Morphological comparison between *C. nebulosa* and other species of *Calamaria* distributed in Yunnan Province are summarised below. *C. nebulosa* differs from *C. yunnanensis* in the following characters: (1) a relatively large number of ventrals in males (179-192 vs. 167-189) and females (198-206 vs. 189-199); (2) longer total length in males (302.5-354 vs. 245-300); (3) and shorter total length in females (272-381 vs. 435-516). *C. nebulosa* differs from *C. andersoni* in the following characters: (1) absent preocular (vs. present); (2) more ventrals in males (179-192 vs. 161-171) and females (198-206 vs. 186). *C. nebulosa* differs from *C. synergis* in the following characters: (1) absent preocular (vs. present); (2) six scales surrounding paraparietal (vs. five); (3) nine maxillary teeth (vs. eight); (4) rostral height less than width (vs. greater than); (5) dorsum with pale light nuchal collar or absent (vs. present light); (6) more ventrals in males (179-192 vs. 161-166). *C. nebulosa* differs from *C. pavimentata* in the following characters: (1) absent preocular (vs. present); (2) rostral height less than width (vs. greater than or equal); (3) posterior chin-shields meeting only in anteriorly (vs. meeting in mid-line ); (4) Obtusely pointed tail tip (vs. sharply pointed).

#### Notes

##### Etymology

According to the meaning of *nebulosa* and English common name clouded reed snake, we propose the following Chinese name “云雾两头蛇” (yún wù liǎng tóu shé).

##### Distribution 

*C*alamaria* nebulosa* is currently known from Phongsaly District, Phongsaly City, Phongsaly Province, Laos; and Simao District, Pu'er City, Yunnan Province, China.

##### Ecology

*C. nebulosa* is a terrestrial, nocturnal species. In our case, individuals of this species were collected on an asphalt rural road in a low hilly landscape with terraced farmlands in the morning (Fig. 5). The local vegetation comprises eucalyptus, abundant herbaceous grasses and shrubs, as well as evergreen broadleaf forest. The microhabitat is crossed by an asphalt rural road with moderate anthropogenic disturbance from vehicle traffic and agricultural reclamation. The soil type is mountain red earth. The climate is humid subtropical, with moist air and often overcast conditions.

##### Conservation status

Based on the limited material of only four *C. nebulosa* specimens, the population size and genetic diversity cannot be evaluated at present. We provisionally assess *C. nebulosa* as Data Deficient (DD) under the IUCN Red List criteria. This assessment is, therefore, preliminary and additional field surveys, specimens and molecular data are required to re-evaluate its threat status in the future.

## Identification Keys

### Identification key to *Calamaria* species from India, China and mainland Southeast Asia.

**Table:** 

1	Preocular absent	2
–	Preocular present	5
2	Subcaudals 16–21 (female), 15–28 (male)	3
–	Subcaudals 10–12 (female), 14–20 (male); ventrals 215–249 (female)	*C. lovii*
3	Subcaudals 15–23 (male), 16–19 (female)	4
–	Subcaudals 28 (male), 21 (female); ventrals 184 (male), 198 (female)	*C. thanhi*
4	ventrals 179–192 (male), 198–206 (female); Subcaudals 22–23 (male), 17 (female)	*C. nebulosa*
–	ventrals 167–184 (male), 189–199 (female); Subcaudals 15–21 (male), 16–19 (female)	*C. yunnanensis*
5	Supralabials 5	6
–	Supralabials 4 (or not counted)	11
6	Dorsum with a light nuchal collar	7
–	Nuchal collar absent	8
7	Ventrals 143–144 (male), 147–162 (female); subcaudals 21–22 (male), 15–19 (female)	*C. albiventer*
–	Ventrals 126–132 (males), 142–144 (female); subcaudals 31–32 (male), 24–25 (female)	*C. prakkei*
8	Dorsal scales with light spots absent	9
–	Dorsal scales with light spots present	*C. buchi*
9	Ventrals ≤ 196 (male), ≤ 229 (female)	10
–	Ventrals 209 (male); total length 578 mm	*C. concolor*
10	Tail ending in a sharp point; subcaudals 17–27 (male), 13–21 (female)	*C. lumbricoidea*
–	Tail not ending in a sharp point; subcaudals 25–44 (male), 19–37 (female)	*C. schlegeli*
11	Dorsum with a light nuchal collar	12
–	Nuchal collar absent or indistinct	22
12	Dorsal light spots absent	13
–	Dorsal light spots present; subcaudals 6–11 (female), 15–19 (male)	*C. septentrionalis*
13	Tail without light rings	14
–	Tail with light rings; subcaudals 15 (female), 20–23 (male)	*C. similis*
14	Scales surrounding paraparietal 5 or 5–6	15
–	Scales surrounding paraparietal 6	17
15	paraparietal 5–6, rostral height < width	16
–	paraparietal 5, rostral height > width	*C. synergis*
16	Ventrals 190 (males and females); subcaudals 19 (male and female)	*C. sangi*
–	Ventrals 180 (female); subcaudals 13 (female)	*C. flavicollaris*
17	Tail length/Total length ≤ 6.5% (female or unknown)	18
–	Tail length/Total length 10.3–13.1%, (female), 14.2% (male); 166-175 (female), 147-155 (male); subcaudals 13-15 (female), 22-27 (male)	*C. garoensis*
18	Posterior chin shields diverging or meeting only anteriorly	19
–	Posterior chin shields meeting in mid-line	*C. berezowskii*
19	Tail not ending in a sharp point	20
–	Tail ending in a sharp point	*C. hunanensis*
20	Rostral height < width	21
–	Rostral height > width; Ventrals 166–175 (female), 147–155 (male)	*C. mizoramensis*
21	Ventrals 196–204 (female), 182 (male); 14-15 (female), 22 (male)	*C. annamensis*
–	Ventrals 170 (male); subcaudals 21 (male)	*C. incredibilis*
22	Dorsal scales with light spots present	23
–	Dorsal scales with light spots absent	24
23	Tail ending in a sharp point; no light rings; ventrals 159 (males), 174 (female)	*C. abramovi*
–	Tail ending in an obtuse point; with light rings; ventrals 170–176 (male), 192 (female)	*C. arcana*
24	Tail without light rings	25
–	Tail with light rings; ventrals 191 (male); total length 457 mm	*C. gialaiensis*
25	Tail ending in a sharp point	26
–	Tail not ending in a sharp point	27
26	Subcaudals 29–33 (male), 20–30 (female)	*C. strigiventris*
–	Subcaudals 13–33 (male), 8–20 (female)	*C. pavimentata*
27	Supralabials 4	28
–	Supralabials 4–5; subcaudals 17–18 (female); ventrals 174 (female); TaL/TL 6.2 (female)	*C. dominici*
28	Tail length/Total length 5.8% (female), 8.0–9.2% (male); ventrals 166–171 (male), 186 (female); subcaudals 14 (female); subcaudals 21-23 (male)	*C. andersoni*
–	Tail length/Total length 3.6–4.6% (female), 7.1–10.1% (male); ventrals 158–160 (male), 177–179 (female)	*C. jinggangensis*

## Analysis

### Sequences

The new molecular sequences have been deposited in GenBank under accession numbers PZ399355–PZ399357. The final alignment of the mitochondrial cyt *b* dataset was 1087 bp for four *C. nebulosa* individuals; 1072 sites were conserved, 15 sites were variable and five sites were parsimony informative. The average nucleotide composition was as follows: 32.3% (A), 28.7% (T), 28.9% (C) and 10.2% (G).

### Genetic distances

The uncorrected *p*-distances of the cyt *b* gene amongst the examined species of *Calamaria* are listed in Suppl. material 3. Intraspecific uncorrected *p*-distances within *C. nebulosa* varied from 0.00% to 1.30%. Interspecific uncorrected *p*-distances between *C. nebulosa* and other species of *Calamaria* ranged from 14.15%-21.35%.

### Molecular phylogeny

The best-fit substitution models for the molecular dataset was TVM+F+I+G4. ML and BI constructed largely congruent topologies. All sampled species of *Calamaria* cluster into three well-supported major clades. Phylogenetic relationships within major clades, including A (97/1.0), B (99/1.0), C (100/1.0) and D (85/0.97) were consistently recovered in accordance with previous work (Bharali et al. 2026, Aksornneam et al. 2026, Qi et al. 2026b). Three of the newly-collected specimens formed a highly supported clade with the holotype, which was clustered with other *Calamaria* species of Clade C (Fig. 6).

## Discussion

Several *Calamaria* species were originally described, based on a single specimen or only one sex, such as *C. gialaiensis* Ziegler, Sang & Truong, 2008, *C. dominici* Ziegler, Tran & Nguyen, 2019, *C. incredibilis* and *C. hunanensis* etc (Ziegler et al. 2008, Ziegler et al. 2020, Qi et al. 2026b, Zhang et al. 2026). Thus, expanded morphological re-descriptions of these species are essential. Prior to *C. nebulosa* being discovered, only one female specimen had been known (Lee 2021). Herein, we collected new specimens of *C. nebulosa* from Yunnan, China.

Morphological examination revealed that the newly-collected specimens of *C. nebulosa* largely agree with the holotype and can be distinguished from other species of *Calamaria* by diagnostic morphological features (Lee 2021). Owing to individual, sexual or age, measurable indices including snout-vent length, tail length and total length show differences amongst individuals. Major differences between the holotype and the three newly-collected specimens include tail length to total length, ventrals, subcaudals and dorsal colour. However, the aforementioned morphological differences may also be attributed to geographic variation.

Phylogenetic analysis and genetic distance data strongly confirm that the newly-collected specimens are conspecific with *C. nebulosa*. Compared with previous studies, our phylogenetic tree placed *C. nebulosa* at the basal position of Clade C (Indochina, China and East Asian islands). In addition, *C. hunanensis* forms a clade with *C. incredibilis* instead of *C. nebulosa*, a result consistent with uncorrected *p*-distances (Zhang et al. 2026). Besides, phylogenetic reconstructions, based on one mitochondrial gene cyt b, may produce unstable topologies. Additional mitochondrial and nuclear genetic markers will be required in future studies to further verify interspecific relationships within the genus *Calamaria*.

Notably, *C. hunanensis* and *C. incredibilis* exhibit genetic differentiation (*p*-distance = 4.39%) and are sister species. *C. hunanensis* differs from *C. incredibilis* in the following characters: (1) more ventrals (181 vs. 170); (2) more subcaudals (23 vs. 21); (3) sharply pointed tail tip (vs. obtusely pointed); (4) two thin and faint brown longitudinal stripes on both sides of the dorsal mid-line, extending from the nuchal collar to the tail tip (vs. a single bold brown stripe along the dorsal mid-line). In addition, the specimen GEP r093 is more similar to *C. hunanensis* in molecular and morphological characters. We, therefore, suggest to re-assign the specimen GEP r093 to *C. hunanensis*.

To date, a total of twelve *Calamaria* species are documented from China, including *C. arcana*, *C. andersoni* Yang & Zheng, 2018, *C. berezowskii* Günther, 1896, *C. hunanensis*, *C. pavimentata*, *C. incredibilis*, *C. jinggangensis*, *C. nebulosa*, *C. septentrionalis* Boulenger, 1890, *C. similis*, *C. synergis* and *C. yunnanensis* (Qi et al. 2026b, Zhang et al. 2026).

## Supplementary Material

XML Treatment for Calamaria nebulosa

0F8DF039-94EA-56E4-BC89-77DC1D298B09 10.3897/BDJ.14.e217560.suppl1 Supplementary material 1 Information of partial *Calamaria* species and outgroups Data type Table Brief description Information of partial *Calamaria* species and the outgroups used in this study. “-” indicates no data. File: oo_1821498.docx https://binary.pensoft.net/file/1821498 Guocheng Shu, Chunmei Fu, Jun Huang, Zhongping He, Cheng Li, Peng Guo, Bingjun Dong, Shun Ma

ACB635DC-F0DD-5393-A1F1-1A95AA09CE5B 10.3897/BDJ.14.e217560.suppl2 Supplementary material 2 Measured morphological characteristics and abbreviations Data type Abbreviations File: oo_1813268.docx https://binary.pensoft.net/file/1813268 Guocheng Shu, Chunmei Fu, Jun Huang, Zhongping He, Cheng Li, Peng Guo, Bingjun Dong, Shun Ma

466B6F05-B3B4-561E-AD8C-463DDB0AB8DB 10.3897/BDJ.14.e217560.suppl3 Supplementary material 3 Uncorrected p-distances Data type Table Brief description Uncorrected p-distances (percent) amongst *Calamaria* species calculated from the mitochondrial cyt *b* gene. File: oo_1816197.docx https://binary.pensoft.net/file/1816197 Guocheng Shu, Chunmei Fu, Jun Huang, Zhongping He, Cheng Li, Peng Guo, Bingjun Dong, Shun Ma

## Figures and Tables

**Figure 1.**
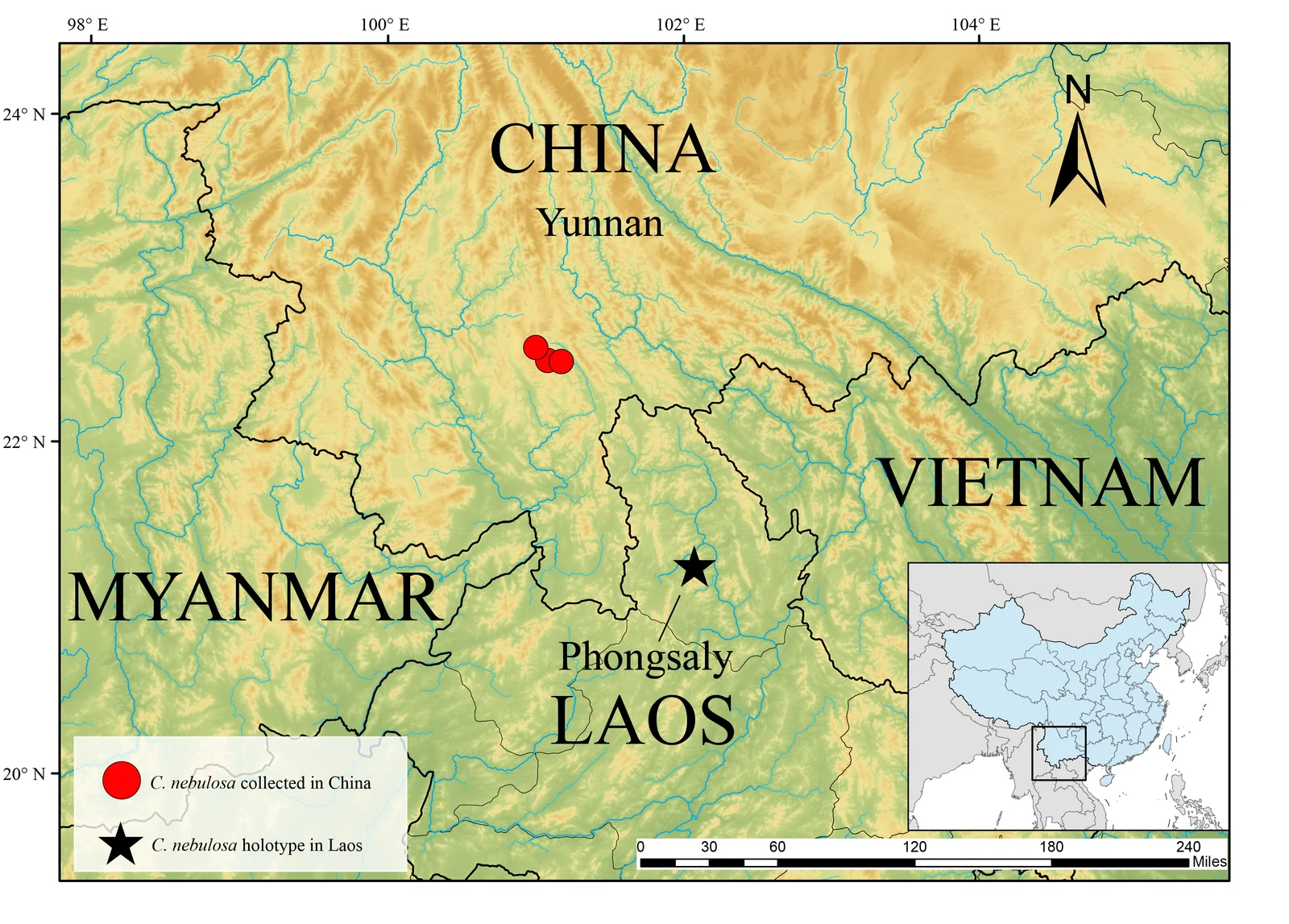
Geographic distribution of *C. nebulosa* in China and Laos. The black star represents the holotype in Phongsaly City, Phongsaly Province and the red circles indicate the new distribution in Simao District, Pu'er City, Yunnan Province.

**Figure 2.**
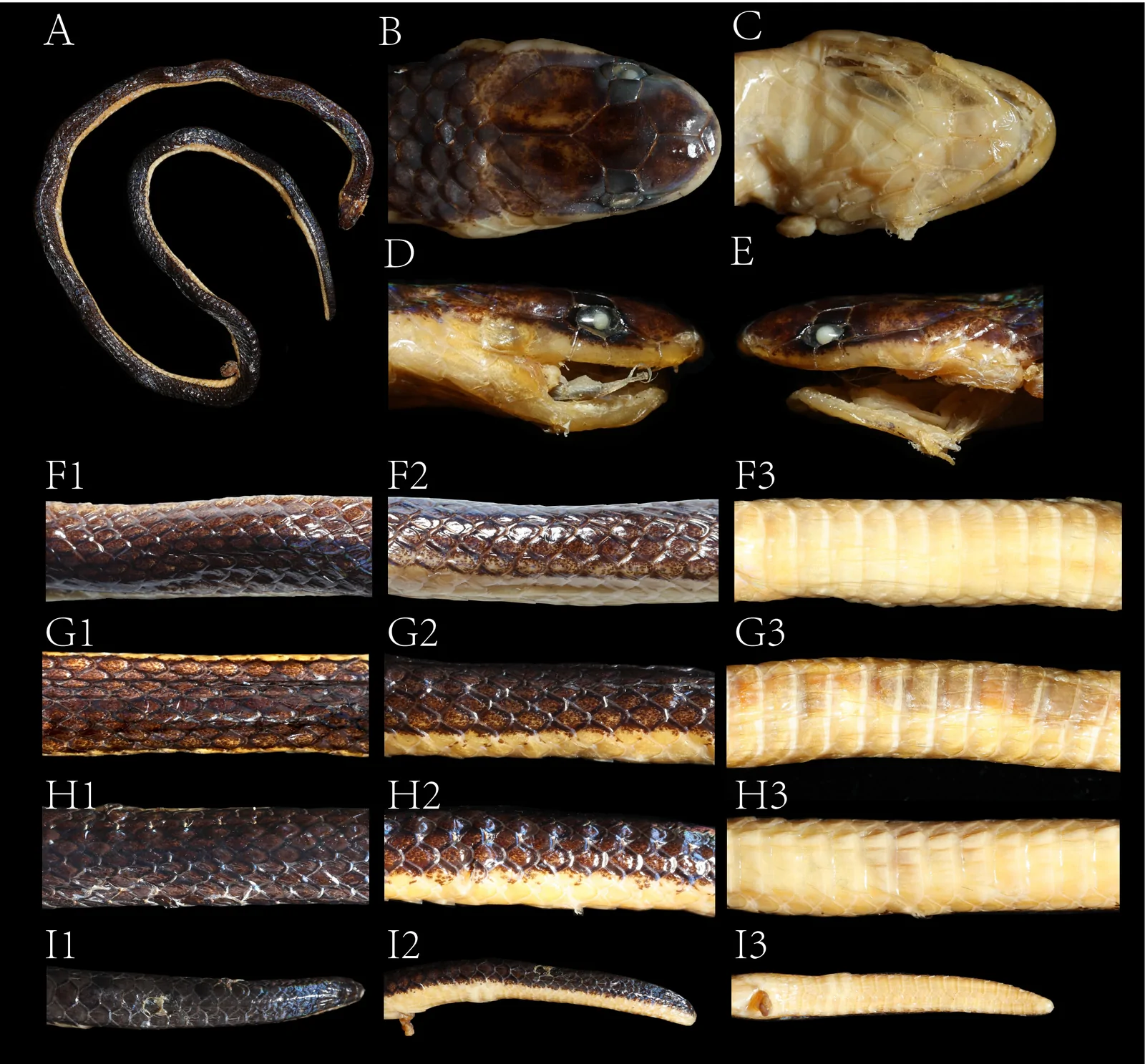
Preserved specimen of *C. nebulosa* (YBU-MS856, male). **A** Dorsal view of body; **B** Dorsal view of head; **C** Ventral view of head; **D** Right lateral view of head; **E** Left lateral view of head; **F** Anterior view of body; **G** Mid-body view of body; **H** Posterior view of body; I. view of tail. 1. Dorsal view; 2. Lateral view; 3. Ventral view.

**Figure 3.**
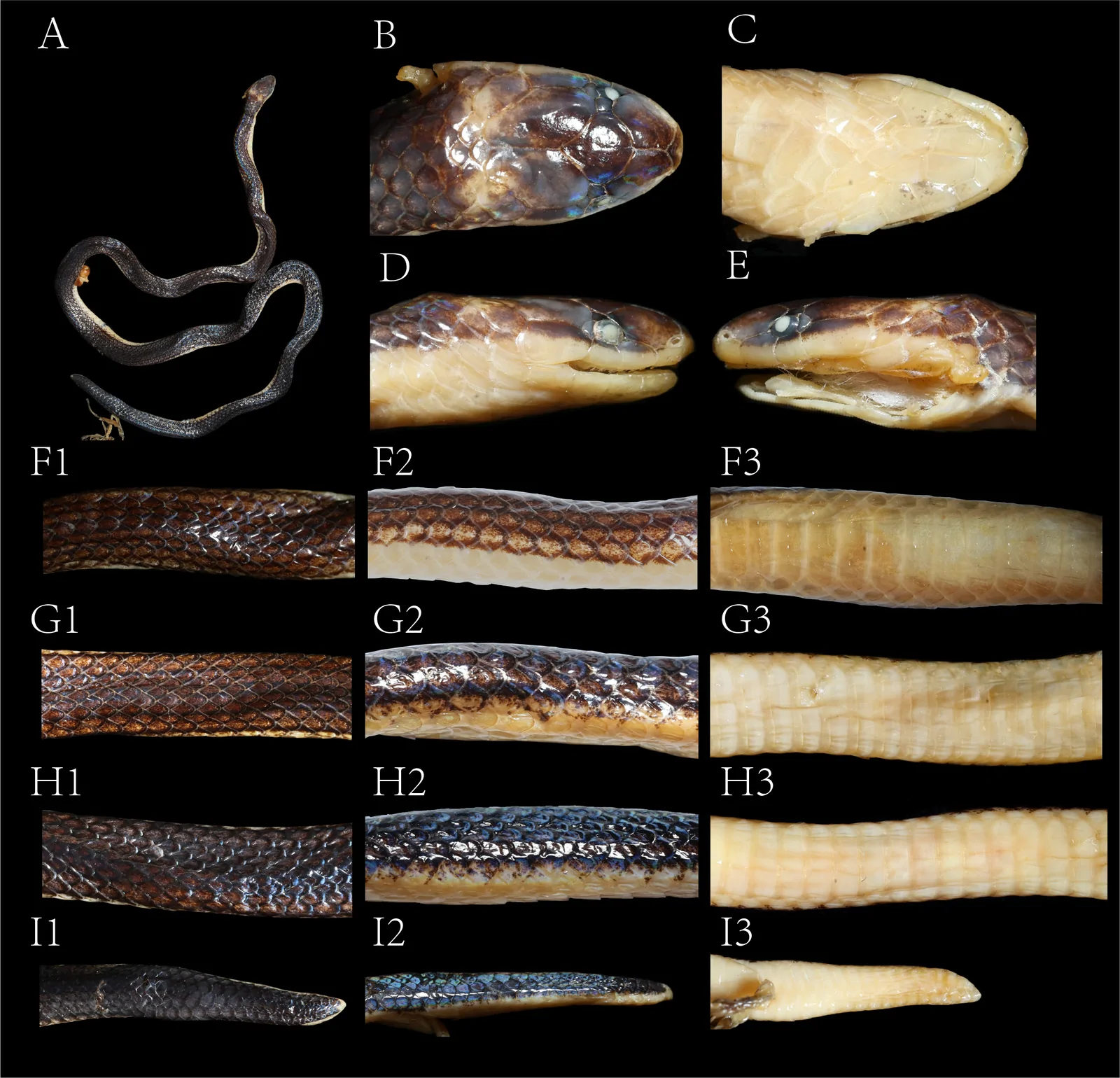
Preserved specimen of *C. nebulosa* (YBU-MS857, female). **A** Dorsal view of body; **B** Dorsal view of head; **C** Ventral view of head; **D** Right lateral view of head; **E** Left lateral view of head; **F** Anterior view of body; **G** Mid-body view of body; **H** Posterior view of body; **I** view of tail. 1. Dorsal view; 2. Lateral view; 3. Ventral view.

**Figure 4.**
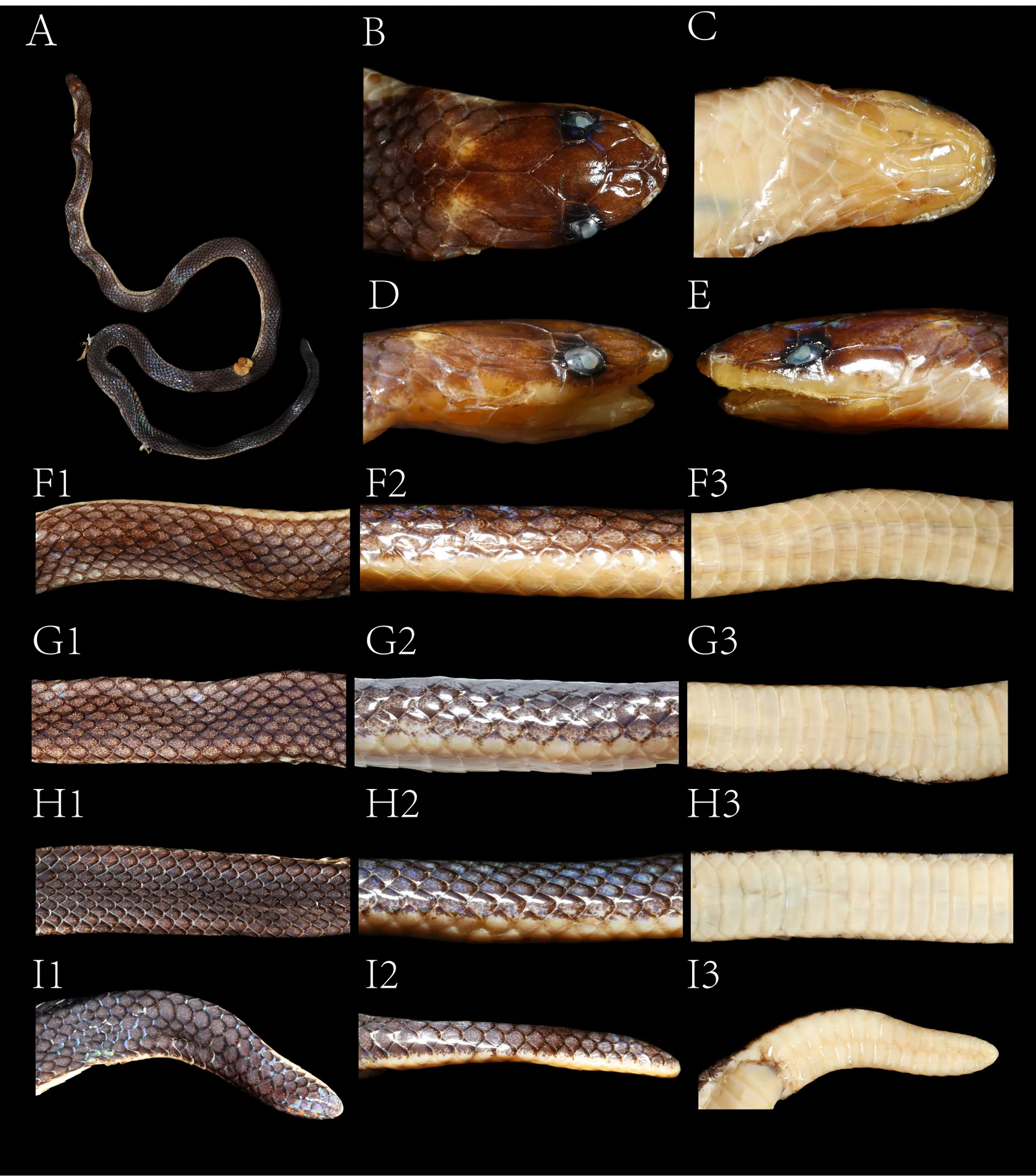
Preserved specimen of *C. nebulosa* (YBU-MS858, female). **A** Dorsal view of body; **B** Dorsal view of head; **C** Ventral view of head; **D** Right lateral view of head; **E** Left lateral view of head; **F** Anterior view of body; **G** Mid-body view of body; **H** Posterior view of body; **I** view of tail. 1. Dorsal view; 2. Lateral view; 3. Ventral view.

**Figure 5.**
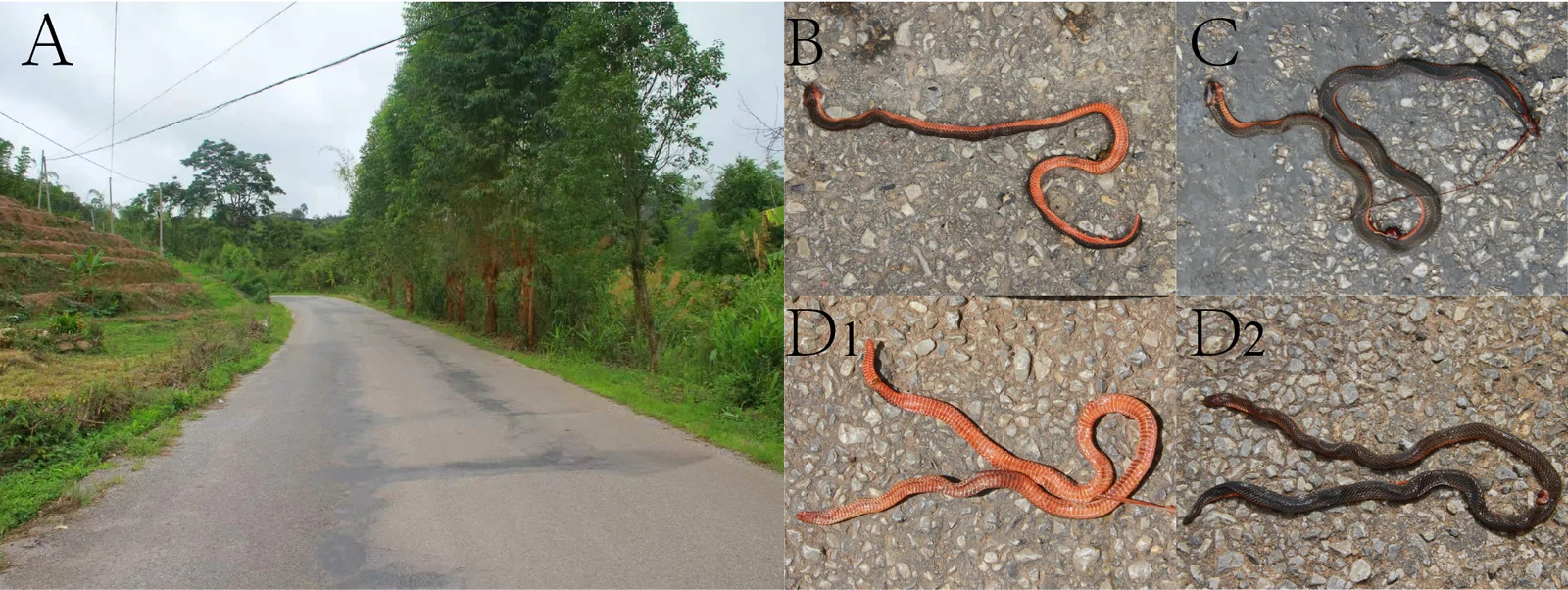
**A**. Habitat at the collecting locality. **B-D**. Road-kill photographed in situ; **B**. YBU-MS856; **C**. YBU-MS857; **D**. YBU-MS858.

**Figure 6.**
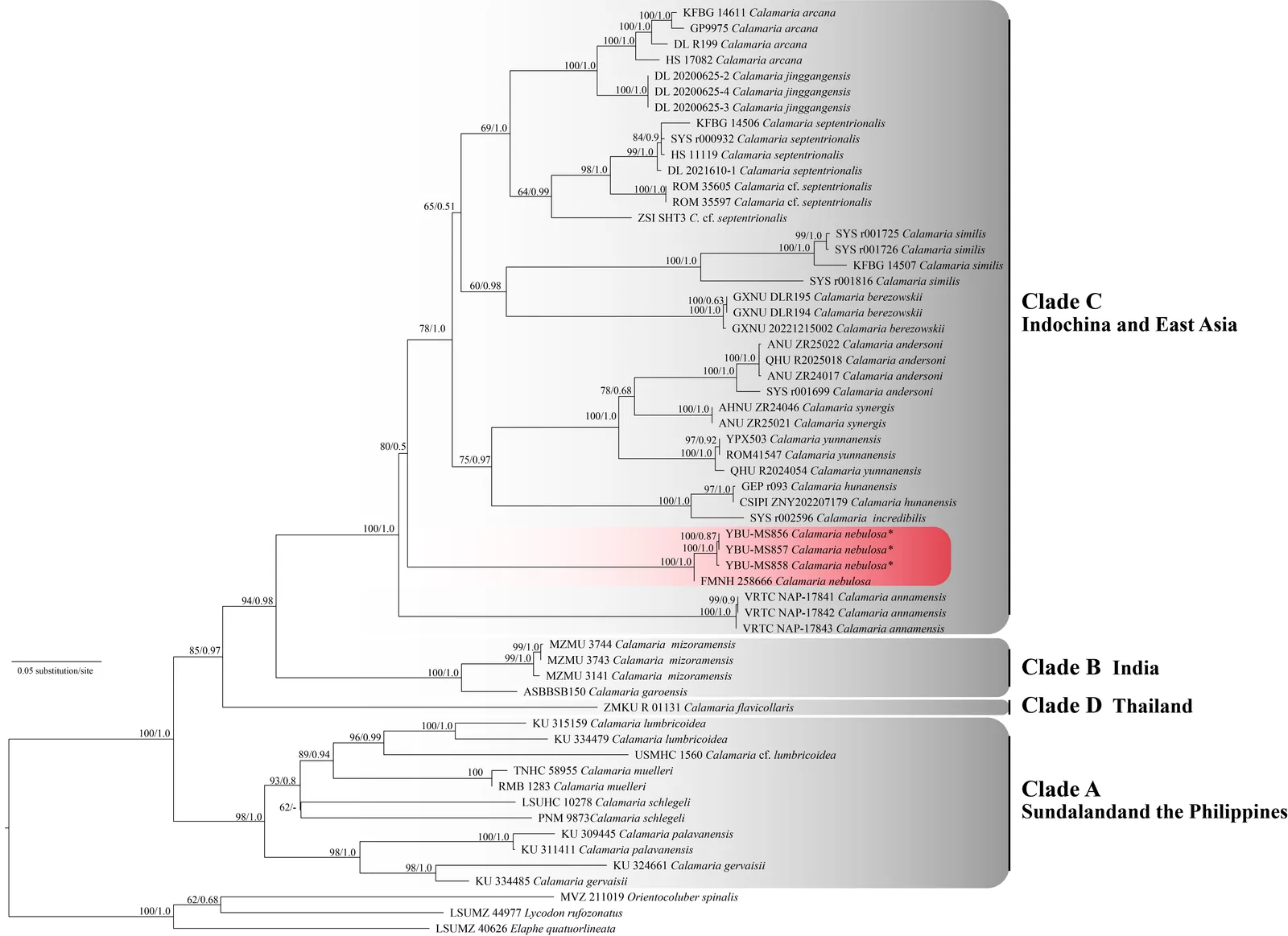
BI and ML phylogenetic trees of *Calamaria* reconstructed, based on the mitochondrial gene cyt *b*. Node values indicate BS and PP support values.

**Table 1.** Morphological comparisons between holotype (FMNH258666) and collected specimen. L = length; W = width. Abbreviations are noted in the materials and methods. “-” indicates no data. Units of length are in mm.

Morphological character	C. nebulosa	C. nebulosa	C. nebulosa	C. nebulosa
Museum catalogue	FMNH258666	YBU-MS856	YBU-MS857	YBU-MS858
Locality	Phongsaly, Laos	Simao, Yunnan, China	Simao, Yunnan, China	Simao, Yunnan, China
Sex	F	M	F	F
SVL	326	275.5	256	359.7
TailL	28	27	16	21.5
TotalL	354	302.5	272	381.2
TailL/TotalL (%)	7.9	8.9	5.9	5.6
HeadW	5.6	6.05	4.86	5.70
HeadL	8.8	8.13	6.82	8.10
HeadH	-	1.88	2.53	2.46
EyeD	1.3	1.1	0.9	1.1
SnL	2.8	2.9	2.5	3.1
SnW	2.1	2.2	1.6	1.8
NarEye	2	1.7	1.5	1.9
IOD	3.5	3.3	2.7	3.3
HeadL/W	1.58	1.34	1.40	1.42
HeadL/SVL	0.027	0.030	0.027	0.023
SnL/HeadL	0.32	0.35	0.37	0.38
EyeD/SnL	0.46	0.37	0.38	0.35
NarEye/SnL	0.71	0.60	0.61	0.62
SnW/IOD	0.60	0.67	0.59	0.55
IOD/HW	0.63	0.54	0.56	0.57
RostralW	2.0	1.88	1.53	1.79
RostralH	1.6	0.75	1.03	1.13
ParietalsW	-	2.07/2.08	1.74/1.88	2.12/2.04
ParietalsL	4.0	3.40/ 3.46	3.59/3.40	3.80/3.93
Suture length of parietals	2.5	1.99	2.06	2.14
PrefrontalsW	-	1.90/1.89	1.65/1.65	2.06/2.04
PrefrontalsL	2.3	2.13/2.18	2.32/2.22	2.56/2.66
Suture length of prefrontals	1.8	1.21	1.14	1.08
FrontalW	1.9	1.92	2.24	2.25
FrontalL	2.7	2.55	2.37	2.73
Rostral	1	1	1	1
Nasals pair	1	1	1	1
Prefrontals pair	1	1	1	1
Frontal	1	1	1	1
Parietals pair	1	1	1	1
Paraparietal	1/1	1/1	1/1	1/1
Scales around paraparietal	6/6	6/6	6/6	6/6
Supraoculars	1/1	1/1	1/1	1/1
Postoculars	1/1	1/1	1/1	1/1
Preocular	0/0	0/0	0/0	0/0
Supralabials	4/4	4/4	4/4	4/4
Infralabials	5/5	5/5	5/5	5/5
Mental	1	1	1	1
Ventrals	179	192	198	206
Anal scale	1	1	1	1
Anal divided (0) or not (1)	1	1	1	1
Dorsals	13–13–13	13–13–13	13–13–13	13–13–13
Subcaudals	22	23	17	17
Total body scales	202	217	222	228
Subcaudal/ventrals	0.109	0.106	0.077	0.075
DSR8R	-	1st SC	1st SC	1st SC
DSR6R	12th SC	4th SC	4th SC	4th SC
DSR4R	22nd SC	20th SC	14th SC	16th SC
End of tail	Obtuse point	Obtuse point	Obtuse point	Obtuse point
Dorsal longitudinal stripes	5	5	5	5
Margins of ventrals	Immaculate	Immaculate	Immaculate	Immaculate
Portion of rostral scale visible from above greater than half with length of prefrontal suture (1) or less than or equal to half its length (0)	0	0	0	0
Prefrontal: longer than frontal scale (1) or smaller than/equal to frontal scale (0)	0	0	0	0
Pre-ocular: present (1) or absent (0)	0	0	0	0
Mental touching (1) or separated from (0) anterior genitals	0	0	0	0
Posterior chin-shields meeting in mid-line (1), diverging or meeting only anteriorly (0)	0	0	0	0
Dorsum colouration in preservative	Bluish-grey	Dark-brown	Dark-brown	Dark-brown
Venter colouration in preservative	Yellow	Yellow	Yellow	Yellow
Dorsal scales with light spots present (1) or absent (0)	0	0	0	0
Dorsum with light nuchal collar (1) or absent (0)	0	0	0	0
Dorsum with light occipital collar (1) or absent/indistinct (0)	0	0	0	0
Tail with rings: present (1) or absent (0)	0	0	0	0
Underside of tail with dark mid-ventral stripe present (1) or absent (0)	0	0	0	0
Source	Lee (2021)	This study	This study	This study
