# Supplementary material for: The first record of Calamaria nebulosa Lee, 2021 (Squamata, Calamariidae) from Yunnan Province, China and an expanded morphological description based on newly-collected specimens
Source: Biodivers Data J. 2026 Nov 5;14:e217560. doi: 10.3897/BDJ.14.e217560 (PMC13760344; doi:10.3897/BDJ.14.e217560)
Supplement: Supplementary file 1 [file bdj-14-e217560-s001.docx]

**Supplementary file 1.** Information of partial *Calamaria* species and the outgroups used in this study. “-” indicates no data.

| No. | Species | Specimen voucher | Locality | Cyt b | Sources |
| --- | --- | --- | --- | --- | --- |
| 1 | *C. nebulosa* | YBU-MS856 | Simao, Yunnan, China | PZ399355 | This study |
| 2 | *C. nebulosa* | YBU-MS857 | Simao, Yunnan, China | PZ399356 | This study |
| 3 | *C. nebulosa* | YBU-MS858 | Simao, Yunnan, China | PZ399357 | This study |
| 4 | *C. nebulosa* | FMNH 258666 | Phongsaly, Laos | MN338524 | Lee 2021 |
| 5 | *C. alcalai* | PNM 9873 | Sablayan, Mindoro, Philippines | MT819383 | Weinell et al. 2021 |
| 6 | *C. andersoni* | SYS r001699 | Yingjiang, Yunnan, China | MH445955 | Yang and Zheng 2018 |
| 7 | *C. andersoni* | ANU ZR25022 | Mangshi, Dehong, Yunnan, China | PV745123 | Zhang et al. 2025 |
| 8 | *C. andersoni* | QHU R2025018 | Mangdong, Dehong, Yunnan, China | PV915792 | Zhang et al. 2025 |
| 9 | *C. andersoni* | ANU ZR24017 | Mangdong, Lianghe, Dehong | PV915791 | Zhang et al. 2025 |
| 10 | *C. annamensis* | VRTC NAP-17841 | Bac Huong Hoa NR, Quang Tri, Vietnam | PX607279 | Korolev et al. 2026 |
| 11 | *C. annamensis* | VRTC NAP-17842 | Bac Huong Hoa NR, Quang Tri, Vietnam | PX607280 | Korolev et al. 2026 |
| 12 | *C. annamensis* | VRTC NAP-17843 | Bac Huong Hoa NR, Quang Tri, Vietnam | PX607281 | Korolev et al. 2026 |
| 13 | *C. arcana* | KFBG 14611 | Mt. Dadongshan, Guangdong, China | ON482335 | Yeung et al. 2022 |
| 14 | *C. arcana* | HS 17082 | Mt. Dawu, Guangdong, China | OQ354835 | Cai et al. 2023 |
| 15 | *C. arcana* | GP 9975 | Yongxing, Hunan, China | OP980549 | Cai et al. 2023 |
| 16 | *C. arcana* | DL R199 | Mt. Wuyi, Fujian, China | OQ354834 | Cai et al. 2023 |
| 17 | *C. berezowskii* | GXNU DLR194 | Mt. Gongga, Sichuan, China | PP747047 | Liang et al. 2024 |
| 18 | *C. berezowskii* | GXNU DLR195 | Mt. Gongga, Sichuan, China | PP747048 | Liang et al. 2024 |
| 19 | *C. berezowskii* | GXNU 20221215002 | Mt. Gongga, Sichuan, China | PP747049 | Liang et al. 2024a |
| 20 | *C. flavicollaris* | ZMKU R 01131 | Suan Phueng, Ratchaburi, Thailand | PZ351576 | Aksornneam et al. 2026 |
| 21 | *C. garoensis* | ADBUSB150 | Oragitok, West Garo Hills, Meghalaya, India | PX645168 | Bharali et al. 2026 |
| 22 | *C. gervaisii* | KU 324661 | Puguis, La Trinidad, Benguet, Luzon,Philippines | MT819384 | Weinell et al. 2021 |
| 23 | *C. gervaisii* | KU 334485 | Narvacan, Ilocos Sur, Luzon, Philippines | MT819385 | Weinell et al. 2021 |
| 24 | *C. hunanensis* | CSIPI | Suining, Shaoyang, Hunan, China | PZ322771 | Zhang et al. 2026 |
| 25 | *C. hunanensis* | GEP r093 | Huaping NR, Guilin, Guangxi, China | PZ233954 | Qi et al. 2026b |
| 26 | *C. incredibilis* | SYS r002596 | Nazhi, Aidian, Ningming, Guangxi, China | PZ233955 | Qi et al. 2026b |
| 27 | *C. jinggangensis* | DL 20200625-2 | Mt. Jinggangshan, Jiangxi, China | OQ354831 | Cai et al. 2023 |
| 28 | *C. jinggangensis* | DL 20200625-3 | Mt. Jinggangshan, Jiangxi, China | OQ354832 | Cai et al. 2023 |
| 29 | *C. jinggangensis* | DL 20200625-4 | Mt. Jinggangshan, Jiangxi, China | OQ354833 | Cai et al. 2023 |
| 30 | *C. lumbricoidea* | KU 315159 | Pasonanca NP, Zamboanga, Philippines | MT819388 | Weinell et al. 2021 |
| 31 | *C. lumbricoidea* | KU 334479 | Mt. Lumot, Gingoog, Misamis, Philippines | MT819389 | Weinell et al. 2021 |
| 32 | *C.* cf*. lumbricoidea* | USMHC 1560 | Air Itam Dam, Penang, Malaysia | MN338526 | Quah et al. 2020 |
| 33 | *C. mizoramensis* | MZMU 3141 | Reiek, Mizoram, India | PX557820 | Lalremsanga et al. 2026 |
| 34 | *C. mizoramensis* | MZMU 3143 | Reiek, Mizoram, India | PX557819 | Lalremsanga et al. 2026 |
| 35 | *C. mizoramensis* | MZMU 3144 | Reiek, Mizoram, India | PX557818 | Lalremsanga et al. 2026 |
| 36 | *C. muelleri* | TNHC 58955 | Gowa, South Sulawesi, Indonesia | MT819390 | Weinell et al. 2021 |
| 37 | *C. muelleri* | RMB 1283 | Gowa, South Sulawesi, Indonesia | MT819391 | Weinell et al. 2021 |
| 38 | *C. palavanensis* | KU 309445 | Barangay Irawan, Puerto Princessa, Palawan, Philippines | MT819386 | Weinell et al. 2021 |
| 39 | *C. palavanensis* | KU 311411 | Mt. Mantalingahan, Rizal, Palawan, Philippines | MT819387 | Weinell et al. 2021 |
| 40 | *C. schlegeli* | LSUHC 10278 | Bukit Larut, Perak, Malaysia | MN338525 | Quah et al. 2020 |
| 41 | *C. septentrionalis* | KFBG 14506 | Hainan, China | MH445956 | Yang and Zheng 2018 |
| 42 | *C. septentrionalis* | HS 11119  (CHS 116) | Tunxi, Huangshan, Anhui, China | MK201273 | Li et al. 2020 |
| 43 | *C. septentrionalis* | SYS r000932  (CHS 613) | Mt. Tianjing, Guangdong, China | MK201434 | Li et al. 2020 |
| 44 | *C. septentrionalis* | DL 2021610-1 | Huangsha, Guangxi, China | OQ354838 | Cai et al. 2023 |
| 45 | *C.* cf*. septentrionalis* | ROM 35605 | Phia Oac-Phia Den NP, Cao Bang, Vietnam | AF471081 | Lawson et al. 2005 |
| 46 | *C.* cf*. septentrionalis* | ROM 35597 | Phia Oac-Phia Den NP, Cao Bang, Vietnam | KX694890 | Alencar et al. 2016 |
| 47 | *C.* cf*. septentrionalis* | ZSI_SHT3 | - | MN788502 | - |
| 48 | *C. similis* | KFBG 14507 | Longzhou, Chongzuo, Guangxi, China | MH445957 | Yang and Zheng 2018 |
| 49 | *C. similis* | SYS r001816 | Yangchun, Yangjiang, Guangdong, China | PZ158843 | Qi et al. 2026a |
| 50 | *C. similis* | SYS r001725 | Nonggang NR, Guangxi, China | PZ158841 | Qi et al. 2026a |
| 51 | *C. similis* | SYS r001726 | Nonggang NR, Guangxi, China | PZ158842 | Qi et al. 2026a |
| 52 | *C. synergis* | AHNU ZR24046 | Mt. Jinuo, Xishuangbanna, Yunnan, China | PV745121 | Zhang et al. 2025 |
| 53 | *C. synergis* | ANU ZR25021 | Mt. Jinuo, Xishuangbanna, Yunnan, China | PV745122 | Zhang et al. 2025 |
| 54 | *C. yunnanensis* | ROM 41547 | Simao, Yunnan, China | KX694891 | Zaher et al. 2009 |
| 55 | *C. yunnanensis* | YPX 503 | Yunnan, China | JQ598922 | Grazziotin et al. 2012 |
| 56 | *C. yunnanensis* | QHU R2024054 | Mt. Wanzhangshan, Simao, Yunnan, China | PV755783 | Zhang et al. 2025 |
| 57 | *Orientocoluber spinalis* | MVZ 211019 | Ningxia, China | AY486924 | Nagy et al. 2004 |
| 58 | *Elaphe quatuorlineata* | LSUMZ 40626 | Hungary | AY486931 | Nagy et al. 2004 |
| 59 | *Lycodon rufozonatus* | LSUMZ 44977 | China | AF471063 | Lawson et al. 2005 |

Aksornneam A, Rujirawan A, Yodthong S, Pawangkhanant P, Sung YH, Aowphol A (2026) Integrative taxonomy reveals a newspecies of Calamaria (Squamata: Calamaridae) from the Tenasserim Range, western Thailand. Contributions to Zoology 95: 366-410. https://doi.org/10.1163/18759866-bja10102

Alencar LRV, Quental TB, Grazziotin FG, Alfaro ML, Martins M, Venzon M, Zaher H (2016) Diversification in vipers: phylogenetic relationships, time of divergence and shifts in speciation rates. Molecular Phylogenetics and Evolution 105: 50−62. https://doi.org/10.1016/j.ympev.2016.07.029

Bharali M, Sangma CM, Amarasinghe AAT, Bohra SC, Swargiary P, Marak GC, Hazarika AK, Das M, Asem BM, Lyngdoh J, Lalremsanga HT, Purkayastha J (2026) A new fossorial reed snake (Squamata: Calamariidae: Calamaria) from northeast India, with a nomenclatural synopsis of the Calamaria pavimentata complex. Taprobanica 15(1): 12–25. <https://doi.org/10.47605/tapro.v15i1.397>

Cai B, Jiang JP, Wu YY, Huang S, Fei DB, Ding L (2023) A new species of *Calamaria* (Reptilia: Serpentes: Colubridae) from western Jiangxi Province, China. Russian Journal of Herpetology 30(2): 101–111. https://doi.org/10.30906/1026-2296-2023-30-2-101-111

Grazziotin FG, Zaher H, Murphy RW, Scrocchi G, Benavides MA, Zhang YP Bonatto SL (2012) Molecular phylogeny of the New World Dipsadidae (Serpentes: Colubroidea): a reappraisal. Cladistics 28(5): 437–459. https://doi.org/10.1111/j.1096-0031.2012.00393.x

Korolev AM, Bragin AM, Gorin VA, Le SX, Idiiatullina SS, Xu YH, Zhang TR, Bos C, David P, Nguyen TV, Poyarkov NA (2026) Rediscovery and revalidation of *Calamaria annamensis* Bourret, 1937 (Squamata: Calamariidae), a rare reed snake restricted to the Truong Son Range of central Vietnam. Zootaxa 5752(1): 105–133. https://doi.org/10.11646/zootaxa.5752.1.5

Lawson R, Slowinski JB, Crother BI, Burbrink FT (2005) Phylogeny of the Colubroidea (Serpentes): new evidence from mitochondrial and nuclear genes. Molecular Phylogenetics and Evolution 37(2): 581–601. https://doi.org/10.1016/j.ympev.2005.07.016

Liang YT, Huang ZD, Ding L, Vogel G, Ananjeva NB, Orlov NL, Shi SC, Wu ZJ, Chen ZN (2024) Revalidated after having been described more than a century ago: *Calamaria berezowskii* Günther, 1896 (Squamata: Colubridae) from Sichuan, Southwestern China. Zoosystematics and Evolution 100(3): 897–911. https://doi.org/10.3897/zse.100.125798

Li JN, Liang D, Wang YY, Guo P, Huang S Zhang P (2020) A large scale systematic framework of Chinese snakes based on a unified multilocus marker system. Molecular Phylogenetics and Evolution 148: 106807. https://doi.org/10.1016/j.ympev.2020.106807

Nagy ZT, Lawson R, Joger U, Wink M (2004) Molecular systematics of racers, whipsnakes and relatives (Reptilia: Colubridae) using mitochondrial and nuclear markers. Journal of Zoological Systematics and Evolutionary Research 42(3): 223–233. https://doi.org/10.1111/j.1439-0469.2004.00249.x

Qi S, Nguyen TV, Yang JH, Xu YH, David P, Shi JS, Liu JJ, Rong CZ, Korolev AM, Poyarkov NA, Wang YY (2026) A new species of *Calamaria* (Squamata, Calamariidae) from southern China, previously confused with *Calamaria pavimentata* Duméril, Bibron & Duméril, 1854. Zookeys 1277: 245–280. https://doi.org/10.3897/zookeys.1277.187107

Qi S, Nguyen TV, Wang J, Zeng ZC, Shi JS, Xu YH, Song HM, Huang Z, Mo YM, Poyarkov NA, Wang YY (2026) An unexpected discovery of hidden diversity: a new species of the genus *Calamaria* H. Boie in F. Boie, 1827 (Squamata, Calamariidae) from Guangxi, China. Zoosystematics and Evolution 102(2): 701−718. https://doi.org/10.3897/zse.102.188045

Quah ESH, Anuar MS, Grismer LL, Wood PL, Azizah MN (2020) Systematics and natural history of mountain reed snakes (genus *Macrocalamus*; Calamariinae). Zoological Journal of the Linnean Society 188(4): 1236–1276. https://doi.org/10.1093/zoolinnean/zlz092

Weinell J, Leviton AE, Brown RM (2021) A new species of reed snake, genus *Calamaria* (Colubridae: Calamariinae), from Mindoro Island, Philippines. Philippine Journal of Systematic Biology 14(2): 1–14. https://doi.org/10.26757/pjsb2020b14006

Yang JH, Zheng X (2018) A new species of the genus *Calamaria* (Squamata: Colubridae) from Yunnan Province, China. Copeia 106(3): 485–491. https://doi.org/10.1643/CH-17-663

Yeung HY, Lau MWN, Yang JH (2022) A new species of Calamaria(Squamata: Colubridae) from Guangdong Province, southern China. Vertebrate Zoology 72: 433–444. https://doi.org/10.3897/vz.72.e84516

Zhang MF, Zhu WB, Wei YF, Shu F, Li Q, Gernot V, Niu YD, Ding L, Chen ZN, Guo KJ, Shi JS (2026) A new species of *Calamaria* Boie, 1827 (Squamata: Calamariidae) from Hunan Province, South Central China. Zoological Research: Diversity and Conservation 3(1): 13-24. https://doi.org/10.24272/j.issn.2097-3772.2025.041

Zhang T, Xu Y, Nguyen TV, Poyarkov NA, Peng L, Deng J, Wang X, Huang S (2025) First description of the female of *Calamaria andersoni* Yang & Zheng, 2018 (Squamata: Calamariidae), with an expanded diagnosis. Biodiversity Data Journal 13: e165597. https://doi.org/10.3897/BDJ.13.e165597
