# Supplementary material for: The first record of Calamaria nebulosa Lee, 2021 (Squamata, Calamariidae) from Yunnan Province, China and an expanded morphological description based on newly-collected specimens
Source: Biodivers Data J. 2026 Nov 5;14:e217560. doi: 10.3897/BDJ.14.e217560 (PMC13760344; doi:10.3897/BDJ.14.e217560)
Supplement: Supplementary file 2 [file bdj-14-e217560-s002.docx]

**Measured morphological characteristics and abbreviations**

The following counts were taken:

rostral scale (Rostral), nasal scales (Nasals), prefrontal scales (Prefrontals), frontal scale (Frontal), parietal scales (Parietals), paraparietal scale (Paraparietal), supraocular scales (Supraoculars), postocular scales (Postoculars), preocular scale (Preocular), supralabial scales (Supralabials), infralabial scales (Infralabials), mental scale (Mental), chin-shield, ventral scales (Ventrals), anal scale, gular scales (Gulars), dorsal scales (Dorsals), dorsal scale rows reducing from 13 to 8 (DSR8R), dorsal scales reducing from eight to six rows (DSR6R), dorsal scales reducing from six to four rows (DSR4R), subcaudal scales (Subcaudals).

The average value was calculated:

total length (TotalL), snout-vent length (SVL), tail lengths (TailL), head length (HeadL), head width (HeadW), head height (HeadH), eye diameter (EyeD, measured horizontally between the posterior and the anterior margins of the eye), snout length (SnL, measured from the anterior point of the eye to the tip of the rostral scale), snout width (SnW, measured as a straight-line distance between the medians of the two nostrils), naris-eye distance (NarEye, measured from the anterior point of the eye to the medial point of the nostril), interorbital distance (IOD, the straight-line distance between the eyes at the border of the supraoculars), rostral scale width (RostralW), rostral scale height (RostralH), parietal scales width (ParietalsW), parietal scales length (ParietalsL), prefrontal scales width (PrefrontalsW), prefrontal scales length (PrefrontalsL), frontal scale width (FrontalW), and frontal scale length (FrontalL).
