# Supplementary material for: The first record of Calamaria nebulosa Lee, 2021 (Squamata, Calamariidae) from Yunnan Province, China and an expanded morphological description based on newly-collected specimens
Source: Biodivers Data J. 2026 Nov 5;14:e217560. doi: 10.3897/BDJ.14.e217560 (PMC13760344; doi:10.3897/BDJ.14.e217560)
Supplement: Supplementary file 3 [file bdj-14-e217560-s003.docx]

**Supplementary file 3.** Uncorrected p-distances (percent) among *Calamaria* species calculated from the mitochondrial cytb gene. 1. FMNH 258666; 2. YBU-MS856; 3. YBU-MS 857; 4. YBU-MS858. Notes: n/a = not available.

| Species | | 1 | 2 | 3 | 4 | 5 | 6 | 7 | 8 | 9 | 10 | 11 | 12 | 13 | 14 | 15 | 16 | 17 | 18 | 19 | 20 | 21 | 22 | 23 | 24 |
| --- | --- | --- | --- | --- | --- | --- | --- | --- | --- | --- | --- | --- | --- | --- | --- | --- | --- | --- | --- | --- | --- | --- | --- | --- | --- |
| 1 | *C. nebulosa* | n/a |  |  |  |  |  |  |  |  |  |  |  |  |  |  |  |  |  |  |  |  |  |  |  |
| 2 | *C. nebulosa* | 0.00 | n/a |  |  |  |  |  |  |  |  |  |  |  |  |  |  |  |  |  |  |  |  |  |  |
| 3 | *C. nebulosa* | 0.20 | 0.20 | n/a |  |  |  |  |  |  |  |  |  |  |  |  |  |  |  |  |  |  |  |  |  |
| 4 | *C. nebulosa* | 1.30 | 1.30 | 1.20 | n/a |  |  |  |  |  |  |  |  |  |  |  |  |  |  |  |  |  |  |  |  |
| 5 | *C. alcalai* | 21.35 | 21.35 | 21.05 | 21.35 | n/a |  |  |  |  |  |  |  |  |  |  |  |  |  |  |  |  |  |  |  |
| 6 | *C. andersoni* | 17.84 | 17.89 | 16.76 | 17.61 | 21.77 | 1.28 |  |  |  |  |  |  |  |  |  |  |  |  |  |  |  |  |  |  |
| 7 | *C. annamensis* | 17.35 | 17.33 | 16.22 | 17.24 | 20.00 | 18.33 | 0.06 |  |  |  |  |  |  |  |  |  |  |  |  |  |  |  |  |  |
| 8 | *C. arcana* | 16.46 | 16.46 | 16.32 | 16.35 | 20.13 | 16.60 | 15.81 | 2.47 |  |  |  |  |  |  |  |  |  |  |  |  |  |  |  |  |
| 9 | *C. berezowskii* | 17.69 | 17.69 | 17.68 | 16.67 | 21.05 | 16.07 | 16.46 | 14.92 | 0.19 |  |  |  |  |  |  |  |  |  |  |  |  |  |  |  |
| 10 | *C. flavicollaris* | 20.27 | 20.27 | 19.46 | 19.79 | 21.20 | 19.67 | 20.33 | 18.45 | 19.69 | n/a |  |  |  |  |  |  |  |  |  |  |  |  |  |  |
| 11 | *C. garoensis* | 17.14 | 17.14 | 16.49 | 17.01 | 16.43 | 17.48 | 18.87 | 14.48 | 18.03 | 18.37 | n/a |  |  |  |  |  |  |  |  |  |  |  |  |  |
| 12 | *C. gervaisii* | 19.16 | 19.16 | 19.10 | 18.91 | 15.01 | 19.15 | 20.24 | 17.92 | 19.62 | 18.06 | 16.42 | 7.29 |  |  |  |  |  |  |  |  |  |  |  |  |
| 13 | *C. hunanensis* | 15.19 | 15.18 | 14.81 | 14.80 | 21.13 | 15.77 | 17.00 | 15.29 | 16.63 | 19.46 | 16.33 | 19.40 | 0.10 |  |  |  |  |  |  |  |  |  |  |  |
| 14 | *C. incredibilis* | 15.25 | 15.25 | 14.15 | 15.06 | 21.80 | 14.92 | 17.22 | 14.98 | 16.67 | 20.04 | 17.17 | 19.53 | 4.39 | n/a |  |  |  |  |  |  |  |  |  |  |
| 15 | *C. jinggangensis* | 16.64 | 16.71 | 16.45 | 16.53 | 20.45 | 16.53 | 15.48 | 5.69 | 15.15 | 18.54 | 14.01 | 17.40 | 13.95 | 14.20 | 0.00 |  |  |  |  |  |  |  |  |  |
| 16 | *C. lumbricoidea* | 19.74 | 19.74 | 19.74 | 19.24 | 15.23 | 19.85 | 20.27 | 19.65 | 20.07 | 19.25 | 17.18 | 15.33 | 20.20 | 20.38 | 19.63 | 11.33 |  |  |  |  |  |  |  |  |
| 17 | *C. mizoramensis* | 17.28 | 17.26 | 17.14 | 17.49 | 17.79 | 18.33 | 17.87 | 15.94 | 19.72 | 17.60 | 5.71 | 15.69 | 19.15 | 17.99 | 15.85 | 17.44 | 0.53 |  |  |  |  |  |  |  |
| 18 | *C. muelleri* | 18.98 | 18.98 | 18.98 | 18.17 | 14.85 | 21.20 | 19.25 | 16.92 | 20.50 | 20.46 | 18.04 | 14.66 | 16.55 | 16.55 | 16.02 | 13.77 | 17.09 | 0.81 |  |  |  |  |  |  |
| 19 | *C. palavanensis* | 17.66 | 17.66 | 17.97 | 18.28 | 15.05 | 19.59 | 19.52 | 20.15 | 21.94 | 18.75 | 17.19 | 12.93 | 17.19 | 17.81 | 17.35 | 16.19 | 17.35 | 15.86 | 2.37 |  |  |  |  |  |
| 20 | *C. schlegeli* | 18.40 | 18.38 | 17.95 | 18.11 | 14.74 | 20.30 | 18.85 | 19.43 | 21.17 | 18.83 | 17.55 | 15.40 | 20.07 | 20.11 | 18.94 | 15.46 | 17.95 | 15.75 | 16.11 | n/a |  |  |  |  |
| 21 | *C. septentrionalis* | 17.32 | 17.31 | 17.01 | 16.78 | 21.56 | 14.30 | 15.68 | 10.88 | 14.96 | 19.18 | 15.80 | 18.86 | 14.15 | 14.44 | 10.22 | 20.02 | 17.37 | 19.97 | 18.24 | 19.29 | 1.00 |  |  |  |
| 22 | *C. similis* | 18.23 | 18.23 | 17.59 | 17.92 | 22.14 | 18.33 | 18.08 | 16.36 | 16.02 | 21.34 | 18.91 | 20.40 | 17.14 | 18.23 | 15.97 | 21.34 | 19.94 | 21.53 | 20.28 | 19.63 | 15.44 | 5.67 |  |  |
| 23 | *C. synergis* | 16.82 | 16.81 | 16.15 | 16.43 | 20.75 | 8.76 | 18.35 | 14.74 | 16.57 | 20.75 | 17.41 | 19.70 | 14.94 | 15.25 | 14.76 | 20.65 | 18.67 | 19.25 | 18.59 | 18.66 | 14.05 | 16.62 | 0.00 |  |
| 24 | *C. yunnanensis* | 16.70 | 16.70 | 16.70 | 16.70 | 21.70 | 9.81 | 17.52 | 15.85 | 16.42 | 20.82 | 18.80 | 20.00 | 14.85 | 15.11 | 15.06 | 20.24 | 20.23 | 22.24 | 21.59 | 20.14 | 14.92 | 16.92 | 8.34 | 0.49 |
